# Salvage Radical Prostatectomy for Radio-Recurrent Prostate Cancer: An Updated Systematic Review of Oncologic, Histopathologic and Functional Outcomes and Predictors of Good Response

**DOI:** 10.3390/curroncol28040252

**Published:** 2021-07-29

**Authors:** Bernhard Grubmüller, Victoria Jahrreiss, Stephan Brönimann, Fahad Quhal, Keiichiro Mori, Axel Heidenreich, Alberto Briganti, Derya Tilki, Shahrokh F. Shariat

**Affiliations:** 1 Department of Urology, Medical University of Vienna, 1090 Vienna, Austria; berhard.grubmueller@meduniwien.ac.at (B.G.); Victoria.jahrreiss@meduniwien.ac.at (V.J.); stephan.Broenimann@meduniwien.ac.at (S.B.); fahad.quhal@meduniwien.ac.at (F.Q.); Keiichiro.mori@meduniwien.ac.at (K.M.); 2 Department of Urology, King Fahad Specialist Hospital, Dammam 32253, Saudi Arabia; 3 Department of Urology, The Jikei University School of Medicine, Tokyo 105-8461, Japan; 4 Department of Urology, University Hospital Cologne, 50923 Cologne, Germany; axel.heidenreich@uk-koeln.de; 5 Department of Urology and Division of Experimental Oncology, URI, Urological Research Institute, IRCCS San Raffaele Scientific Institute, 20132 Milan, Italy; briganti.alberto@hsr.it; 6 Department of Urology, University Hospital-Hamburg Eppendorf, 20251 Hamburg, Germany; dtilki@me.com; 7 Karl Landsteiner Institute of Urology and Andrology, 3100 St. Pölten, Austria; 8 Department of Urology, University of Texas Southwestern, Dallas, TX 75390, USA; 9 Department of Urology and Division of Medical Oncology, Weill Medical College of Cornell University, New York, NY 10065, USA; 10 Department of Urology, Second Faculty of Medicine, Charles University, 150 06 Prague, Czech Republic; 11 Institute for Urology and Reproductive Health, I.M. Sechenov First Moscow State Medical University, 119992 Moscow, Russia; 12 Division of Urology, Department of Special Surgery, Jordan University Hospital, The University of Jordan, Amman 11942, Jordan

**Keywords:** prostate cancer, salvage radical prostatectomy, primary radiotherapy, recurrence

## Abstract

A valid treatment option for recurrence after definite radiotherapy (RT) for localized prostate cancer (PC) is salvage radical prostatectomy (SRP). However, data on SRP are scarce, possibly resulting in an underutilization. A systematic review was performed using MEDLINE (Pubmed), Embase, and Web of Science databases including studies published between January 1980 and April 2020. Overall, 23 English language articles including a total number of 2323 patients were selected according to PRISMA criteria. The overall median follow-up was 37.5 months (IQR 35.5–52.5). Biochemical-recurrence (BCR)-free probability ranged from 34% to 83% at five years, respectively, and from 31% to 37% at 10 years. Cancer specific survival (CSS) and overall survival (OS) ranged from 88.7% to 98% and 64% to 95% at five years and from 72% to 83% and 65% to 72% at 10 years, respectively. Positive surgical margins ranged from 14% to 45.8% and pathologic organ-confined disease was reported from 20% to 57%. The rate of pathologic > T2-disease ranged from 37% to 80% and pN1 disease differed between 0% to 78.4%. Pre-SRP PSA, pre-SRP Gleason Score (GS), pathologic stage after SRP, and pathologic lymph node involvement seemed to be the strongest prognostic factors for good outcomes. SRP provides accurate histopathological and functional outcomes, as well as durable cancer control. Careful patient counseling in a shared decision-making process is recommended.

## 1. Introduction

Radiation therapy (RT) is a standard and widely used primary treatment strategy with the intention to cure non-metastatic prostate cancer (PC) [1]. Despite adequate delivery, up to 40% of patients eventually suffer from biochemical recurrence (BCR) [2,3,4]. Despite several studies having shown acceptable oncologic and functional results for salvage radical prostatectomy (SRP) of radio-recurrent PC [5,6,7], most of these patients are still treated with systemic palliative androgen deprivation therapy (ADT) [1,2], with all its downsides, including the very high failure rate and systemic side effects [8,9,10]. A systematic review of the literature published in 2012 by Chade et al. [11] reported on the steadily improving outcomes of SRP resulting from surgical expertise and improved patient selection. Thanks to novel surgical techniques such as the robot-assisted approach [12] and the earlier detection of locoregional recurrent disease with novel superior imaging modalities in the BCR setting (i.e., PSMA-PET) [13], outcomes of salvage local surgical interventions promise to further improve [14]. Most guidelines recommend performing SRP in case of BCR after primary RT (1) in centers with great experience, (2) after confirmatory biopsy of the local relapse, (3) without clinical evidence of metastatic disease [1,15]. The aim of SRP is to cure with local therapy, to delay time to clinical progression and death, to prevent local symptoms, and/or to achieve a local cytoreduction that would allow a better response to subsequent therapies. Additionally, systemic treatment-related toxicity can in some cases be delayed or avoided completely.

Despite progress in this field, exact data on ideal patient selection and predictors of response for SRP is still scarce but urgently needed to help guide clinical decision making for individual patients. Several confounding studies have addressed the oncologic, histopathological, and functional outcomes of SRP over a long period of time. For this reason, we aimed to conduct an updated systematic review of the literature concerning histopathologic and oncologic outcomes for SRP with the aim of identifying predictors of response.

Considering the interventional aim of this meta-analysis, oncologic outcomes (BCR-rate, overall survival (OS), cancer-specific survival (CSS), histopathological outcomes (T stage, N stage, and positive surgical margins (PSM)), as well as functional outcomes (erectile function (EF), urinary continence) have been assessed.

The secondary aim was to compare histopathological outcomes after SRP, the usage of minimally invasive surgery (laparoscopic/robotic) and time to SRP in studies published before and after 2010. Another secondary aim was to compare outcomes of open versus laparoscopic/robotic SRP. Therefore, analyses were conducted among (1) studies published 1988–2009 and 2010–2020, and (2) open vs. laparoscopic/robotic cases.

## 2. Methods

### 2.1. Search Strategy

Databases which are considered the most relevant for our topic will be searched. For this reason, this review is based on systematic searches in the MEDLINE (Pubmed), Embase, and Web of Science databases according to the Preferred Reporting Items for Systematic Reviews and Meta-analysis (PRISMA) guidelines [16]. These electronic databases were searched in April 2020 to identify reports on oncologic, histopathologic, and functional outcomes of SRP for radio-recurrent PC.

Initially, medical subject headings (MeSH) were used followed by free-text terms using the following controlled vocabulary for the further search strategy: “radical AND salvage therapy” OR “salvage AND therapy” OR “salvage therapy” OR “salvage AND prostatectomy” OR “salvage AND prostatectomy AND prostate cancer”. The temporal limit was set to January 1980 and only articles in English were considered for review.

### 2.2. Study Selection

**Figure 1:**
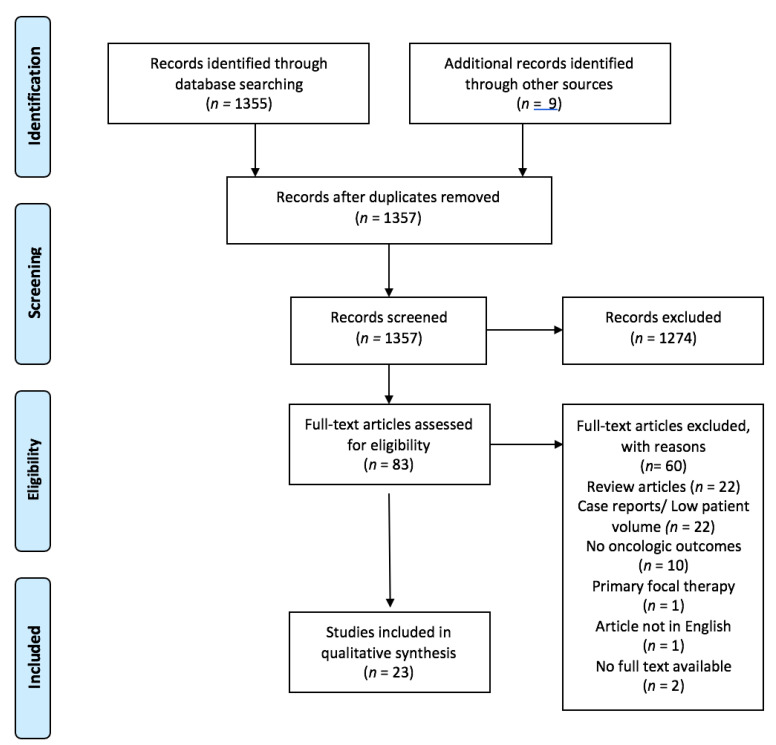
PRISMA flow diagram.

A flow diagram adhering the PRISMA guidelines for reporting of systematic reviews was used in reporting of the selection process and the results (Figure 1).

The initial search results have been organized by importing them into the Endnote reference management software (Thomson Reuters (Scientific) LLC, London, UK). Duplicates and irrelevant studies have been removed. Two independent investigators screened the results based on the titles and abstracts to identify ineligible studies, and reasons for exclusions were noted. Additional references were identified from the reference list of each article. Two independent reviewers subjected potentially relevant reports to a full-text review and the relevance of the reports was confirmed after the data extraction process. Both reviewers had to agree on the inclusion of the study in all cases. In cases of disagreement, a third reviewer was consulted for the final decision. After screening 1364 papers, 23 met the inclusion criteria for synthesis (Figure 1).

### 2.3. Inclusion Criteria

Studies being included had to present data of patients undergoing SRP for radio-recurrent PC, apply a widely accepted qualitative data collection method, and use a well-described methodology.

Criteria for study inclusion took into account the following topics: (1) radio-recurrent PC diagnosis, (2) local recurrence with no evidence of metastatic disease, (3) predictive oncologic factors, (4) surgical approach (open, laparoscopic, or robotic SRP), (5) cancer control, (6) histopathologic outcomes, and (7) functional outcomes.

### 2.4. Exclusion Criteria

We excluded reviews, letters, editorials, meeting abstracts, replies from authors, and case reports with fewer than 10 patients. In the case of duplicate publications, the higher quality or the most recent publication was selected.

### 2.5. Data Extraction

Two investigators independently extracted the following information from the included articles: first author′s name, publication year, recruitment country, period of patient recruitment, number of patients, age, study design, initial PSA, PSA at SRP, TNM stage, surgical technique, oncological outcome, functional outcomes, predictive oncologic factors, and follow-up duration.

### 2.6. Missing Data

In case data could not be acquired, only available data were analyzed.

### 2.7. Statistics

Differences in categorical variables including PSM, organ-confined disease (OCD), T stage, N stage, and usage of laparoscopic/robotic surgery were assessed using Chi-square tests. Continuous variables were analyzed using the t-test. Organ-confined disease was defined as ≤pT2 and N0 disease. All *p* values were two-sided, and statistical significance was defined as a *p* < 0.05. Statistical analyses were performed using Stata/MP 14.2 statistical software (StataCorp., Collage Station, TX, USA).

## 3. Results

### 3.1. Epidemiology

In the absence of RCTs, all 23 studies that reported oncologic outcomes and were included in the analyses were of retrospective design (19 single center [12,17,18,19,20,21,22,23,24,25,26,27,28,29,30,31,32,33,34] and four multi center [5,35,36,37]). The studies were published between 1988 and 2020. These studies comprised data of 2323 patients in total. The median age was reported in all but five studies [5,22,24,30,31]. The median age of the patients at the time of SRP was 65 years (overall interquartile range (IQR) 63.5–65.5). The complete data about primary therapy for localized PC were provided in 18 studies. The percentage of patients receiving ADT as concomitant therapy with RT ranged from 2% to 60% across the studies. Overall, 9.6% of the included 2323 patients underwent RT with concomitant ADT, while the rest of the patients received RT only. The type of RT within the different studies can be seen in Table 1. The exact definition of BCR after primary RT differed between the studies. Nevertheless, all of the included patients had a confirmatory biopsy in case of BCR before SRP. The overall median time to SRP was 51 months (IQR 41–69) and overall median total serum PSA at SRP was 6.04 ng/mL (IQR 4.5–9.4). Information on the surgical technique was provided in 12 studies [5,12,18,19,20,21,22,23,24,31,32,35], compromising 1277 patients. SRP was performed in open approach in 79.9% of the patients, robotic in 19.0%, and laparoscopic in 1.1%. Comparing the surgical technique between studies published before and after 2010, we found a difference in the usage of the laparoscopic/robotic approach over time (0% (0/119) in studies published before 2010 versus 22.4% (259/1158) in studies published after 2010, *p* < 0.0001). We also observed a difference in time to SRP from primary RT over the last decade. While the median time to SRP was 40 months before 2010, it was 68 months after 2010, *p* = 0.0068.

### 3.2. Oncological Outcomes

The studies with the largest number of patients were retrospective and of multicenter design [5,36]. The median follow-up within all studies ranged from 18 to 96 months (overall median follow-up 37.5 months, IQR 35.5–52.5), which may explain the wide variety of findings in oncologic survival outcomes. The definition of BCR after SRP varied between the different publications, although a PSA rise > 0.2ng/mL was the most widely used definition. The five-year BCR-free survival was reported in 10 out of 23 studies and ranged from 34% to 83% [5,12,17,19,22,25,27,33,35,37]. The 10-year BCR-free survival was available in two studies with 31% [23] and 37% [5]. The clinical progression-free survival was reported in only one study [28] with 47% at five years. The CSS was reported in three studies ranging from 88.7% to 98% at five years [5,19,25] and from 72% to 83% [5,31] at 10 years. The reported OS ranged from 64% to 95% at five years [17,25,28,31] and from 65% to 72% at 10 years [28,29,31], respectively.

None of the studies evaluated differences in oncologic SRP outcomes after RT and after brachytherapy (BT) or between distinct RT techniques or RT dose. At the time of SRP, none of the included patients was on ADT. Data evaluating the effect of neoadjuvant or concomitant ADT were poorly described, if at all. Overall, no effect for concomitant ADT at the time of RT was shown on oncologic outcomes after SRP. One study evaluated the impact of concomitant ADT on oncologic outcomes after SRP [31] but found no difference in CSS.

### 3.3. Histopathological Outcomes

The pathologic characteristics after SRP were reported in all but three studies [25,33,34]. Among these studies, several differences in the pathologic outcomes were found. Overall, 847 of the 2323 patients (36.5%) undergoing SRP had a PSM. The PSM-rate ranged from 14% to 45.8%. Pathologic OCD was reported from 20% to 57% and the rate of pathologic > T2-disease ranged from 37% to 80%. Pathologic lymph node (LN) involvement after SRP was reported to be between 0% and 78.4%. Comparing histopathological outcomes between studies published before and after 2010, we could not find a difference in the rate of overall PSM (27.3% vs. 23.9%, *p* = 0.11). Nevertheless, we found a difference in overall pathological LN disease (11.5% in studies before 2010 versus 17.3% in studies published after 2010, *p* = 0.0009) and in the rate of overall ≤pT2-disease after SRP (62.9% in studies before 2010 and 47.4% in studies published after 2010, *p* < 0.0001). Comparing open versus laparoscopic/robotic SRP, there was also no difference in the overall PSM-rate (26.8% vs. 21.8%, *p* = 0.13) and in the pathologic stage after SRP (≤pT2 48.2% vs. 50.5%, *p* = 0.55), but the rate of pathologic N1 disease was higher in the laparoscopic/robotic (31.5%) vs. open cases (15.6%), *p* < 0.0001.

### 3.4. Functional Outcomes

Although the oncologic outcomes of SRP seem to be accurate, sexual and urinary dysfunction after the surgery are directly influencing the patients′ quality of life. Overall, 13 studies (four robotic (216 patients overall) [12,20,21,24], nine open (370 patients overall) [19,23,26,28,30,31,32,33,34]) could be identified reporting on oncologic and functional outcomes after SRP. The outcome measurements and definitions for EF after SRP varied between the different studies: (1) “Sexual Health Inventory for Men” Questionnaire [12,20], (2) IIEF-5-Score [19], (3) “erection sufficient for penetration without PDE-5 inhibitors” [21], (4) “ability to have sexual intercourse with or without the use of PDE-5 inhibitors in >50% of the attempts” [23], (5) “normal erections during intercourse with or without PDE-5 inhibitor” [26]. EF before SRP was already poor with a great variability (two studies did not report on EF-rate before SRP [20,26]) ranging from 25.5% to 40.9%. However, the post-SRP EF-rate dropped significantly and from 0% to 13.1% in five studies [12,19,21,23,26]. One study reported an EF of 31.5% three years after SRP, although the pre-EF-rate was not available [20].

The urinary continence rate after SRP ranged from 21.9% to 90% at one year. Again, here, the definition of urinary continence was not uniform. The definition used most often for urinary continence after SRP was the usage of 0–1 pads/day in eight studies [19,20,21,24,28,31,32,33] and ranged from 27% to 76.9%. Three further studies defined 0 pad/day as urinary continent [23,26,30] and the rate was reported to be between 21.9% and 65%. Onol et al [12] ivided their definition into fully continent (0 pad/day: 73%) and social continent (0–1 pad/day: 39.2%). The study with the highest rate of urinary continence of 90% was published by Rainwater et al. [34], but they did not report their used definition.

### 3.5. Prognostic Risk Factors

Overall, 15 studies could be identified that evaluated both clinical and pathologic predictive factors of oncologic outcomes after SRP [5,17,19,20,21,23,27,28,29,31,32,34,35,36,37]. One included study could not find any association of clinicopathologic factors with oncologic outcome after SRP [20]. Pre-surgical PSA, the pre-surgery Gleason Score (GS) and the pathologic stage including LN status after SRP seemed to be the strongest prognostic factors. Overall, eight studies found an association of pre-surgical PSA with pathologic and oncologic outcomes. A high pre-surgical PSA correlated with BCR in five studies [5,21,27,32,37], with clinical progression and CSS in one study [5], with OS in three studies [27,28,37], and with PSM-rate in one study [36]. Another important pre-operative predictor was the biopsy GS, as it was associated with the BCR [5,17,19,35,37], clinical progression [5], CSS [5,35], and OS [27,28,35,37]. Further pre-operative factors found to be significant were pre-operative clinical stage in two studies [5,28] and the number of positive cores in one study [17]. Concerning post-operative factors, the pathological stage after SRP was associated with BCR in five studies [19,21,23,35,37], with clinical progression in one study [19], with CSS in one study [37] and with OS in two studies [5,28]. Pathologic LN involvement was found to be associated with BCR [19,35,37], clinical progression [5,19], CSS [35], and with OS [5]. Furthermore, pathologic GS was associated with CSS [5,37] and BCR [37]. Another pathologic prognostic risk factor in SRP specimens in older published studies (all before 2000) was DNA ploidy for BCR [29] and CSS [31,34].

## 4. Discussion

Radio-recurrent PC after primary RT with curative intent remains a challenging clinical scenario for physicians given the lack of consensus on patient selection and the fact that standard imaging tools, such as CT and bone scans, are not accurate enough to distinguish between locoregional only, distant recurrence, or both [38]. This is changing with the more widespread use of novel imaging tools such as PSMA-PET imaging in the BCR setting [13]. Due to its superior detection rates (sensitivity) also at lower PSA levels, PSMA-PET allows a more individualized salvage therapy decision making with a potential for cure in cases of locoregional recurrence only [39,40]. This is also the reason why SRP and other potentially curative local treatments are becoming more interesting for the management of radio-recurrent PC, again [41]. Furthermore, as shown by Marra et al., recurrences are frequently high grade, with 27% having a GS ≥9 compared to 90% having a GS of ≤7 at initial PC diagnosis. This discrepancy is likely largely related to treatment induced changes and selection of resistant clones, altering the natural history of PC towards adverse features [42]. Nevertheless, potential risks and side effects and patient selection are still a matter of debate for SRP. The aim of SRP is to cure, to delay progression, to delay the need for systemic therapies, and to prevent local complications. Despite improved early detection of the site of recurrence and despite improved surgical techniques, such as the robot-assisted approach [12], the currently most used treatment for radio-recurrent PC is ADT, which leads to significant morbidity (e.g., bone fractures, diabetes, etc.) in addition to excluding the opportunity for cure [43]. Because of the lack of studies that directly compare SRP to ADT and SRP to nonsurgical local salvage therapies in the case of radio-recurrent PC, we aimed to conduct an updated systematic review of the literature concerning histopathologic and oncologic outcomes for SRP with the aim of identifying predictors of response.

In this systematic review, oncologic outcomes varied among the included studies for the synthesis. Considering a wide range of different follow-up times (from 18 to 96 months) the BCR-free rate ranged from 34% to 83% at five years, meaning that oncological outcomes are promising in the medium term and a significant proportion of men remain free of disease at five years follow-up. The two studies with the largest cohorts [5,36] and the longest follow-up showed a 10-year BCR-free survival of 31% and 37%, respectively. A systematic review and meta-analysis on nonsurgical salvage focal therapies for radio-recurrent PC published in 2020 showed comparable results, but patient selection may account for this [41]. The overall pooled prevalence of BCR-free survival was 64%, with a large heterogeneity within the different studies. In a subgroup analysis, the prevalence of biochemical control was the lowest for patients treated with HIFU (58%) and highest for patients treated with BT (69%) and salvage EBRT (69%). Nevertheless, one has to mention that, in this included meta-analysis, the median follow-up time was comparably shorter, and many patients received adjuvant ADT in addition to salvage nonsurgical focal therapy for radio-recurrent PC. Therefore, a direct comparison is not possible considering the studies included in our work, since none of the patients was on concomitant ADT while undergoing SRP. A meta-regression analysis published in 2016 also compared oncologic outcomes of SRP vs. nonsurgical therapies for radio-recurrent PC [44]. The authors concluded that the oncologic outcomes were comparable between SRP and the nonsurgical salvage modalities. However, SRP was associated with a higher rate of urinary incontinence. In addition, the higher rate of given ADT in the nonsurgical studies prevents a fair direct comparison within the investigated treatment modalities.

Similar oncologic results were published by Valle et al., who compared local salvage modalities (SRP, HIFU, cryotherapy, stereotactic body radiotherapy (SBRT), low-dose-rate (LDR) brachytherapy, and high-dose-rate (HDR) brachytherapy) for radiorecurrent disease. Adjusted 5-yr RFS ranged from 50% after cryotherapy to 60% after HDR brachytherapy and SBRT, with no evidence of large differences in 5-yr RFS outcomes for surgical, non-radiotherapeutic ablative, and radiotherapeutic salvage of radiorecurrent PC. As discussed above, this meta-analysis also included studies with significant heterogeneity between studies and within each modality [45].

Furthermore, the published series on nonsurgical salvage therapies are relatively small and consequently, this treatment should be offered in experienced centers only. Therefore, nonsurgical salvage focal therapies are not recommended by the guidelines, except within a clinical trial setting or well-designed prospective study cohorts [1].

Additionally, we found that CSS in the studies included in our review ranged from 88.7% to 98% at five years and from 72% to 83% at 10 years after SRP. OS ranged from 64% to 95% at five years and from 65% to 72% at 10 years, respectively. Despite these outstanding results, most of the patients with local recurrence after primary RT with curative intent are referred to systemic ADT [1]. Interestingly, one randomized prospective trial showed an 86% vs. 79% OS advantage at 10 years for ADT in this setting [46]. In contrast, most studies did not find any differences between early vs. delayed, or no ADT [47]. Moreover, there are studies showing an even unfavorable effect of ADT on survival due to its long-term risks and side-effect [48]. Nevertheless, some high-risk patients seem to benefit most from early ADT, given a life expectancy of more than ten years [49]. Considering these results, SRP seems to be superior to ADT, concerning survival rates and long-term side effects in well-selected patients in case of radio-recurrent PC after primary RT. ADT should be offered to those patients at highest risk of disease progression since there is supporting data on the improvement of survival [46]. However, these patients are likely to be excluded from SRP with the use of modern scanning such as PSMA-PET nowadays [14].

The overall PSM-rate was 36.5% with a range of 14% to 45.8%. The range of OCD on final SRP pathology ranged from 20% to 57% and the rate of pathologic > T2-disease ranged from 37% to 80%. Overall, 0 –78.4% had pathologic positive LN at the time of SRP. Two of the multi-institutional studies including the highest number of patients [5,36] showed a PSM of 33.7% and 25%, OCD in 50.9% and 53%, and LN involvement in 6.2% and 16%. A systematic review published in 2012 showed similar results [11]. Here, the rate of PSM and of OCD ranged from 0% to 70% and 22% to 81%, respectively. This variety among histopathological findings may be explained due to the long period of time and different centers reporting on SRP outcomes. Because surgical techniques improved over time [50] and the robot-assisted laparoscopic approach became the new standard of care [12] (as we could also show an increased use over the last decade: 0% before 2010, 22.4% after 2010, *p* < 0.0001), we compared the histopathological outcomes in studies published before and after the year 2010. Although we could not find a difference in the rate of overall PSM-rate (*p* = 0.105), there was a difference in overall pathological N1 disease (*p* = 0.0009) and in the rate of overall ≤pT2-disease (*p* < 0.0001) in studies before 2010 compared to those published after 2010. The finding that clinical stage and LN involvement increases over time in SRP specimen can possibly be explained by the introduction and increased use of active surveillance in low-risk PC recurrence and a higher awareness of life expectancy and competing health risks [51]. We also compared the histopathological outcomes of open versus laparoscopic/robotic SRP and found no difference in PSM-rate (*p* = 0.13) and in the pathologic stage after SRP (*p* = 0.55). Nevertheless, the rate of pathologic N1 disease was higher in the laparoscopic/robotic (31.5%) vs. open cases (15.6%), *p* < 0.0001. One has to mention that a limitation of the latter analysis is the scarce available data reporting on outcomes of open vs. laparoscopic/robotic SRP in the literature. One possible explanation for this finding might be the fact that attitudes toward lymphadenectomy changed considerably during the period analyzed [52]. 

Moreover, the functional outcomes differed between the included studies of this systematic review. While the patients had a relatively poor EF before SRP (25.5% to 40.9%), the EF after SRP was reported from 0% to 13.1% in five studies [12,19,21,23,26]. One study reported an EF of 31.5% after three years, but without reporting on the EF before surgery [20]. Clearly, the different definitions and measurements of EF have to be taken into account while interpreting these results. Therefore, the informative value on overall EF after SRP is limited. Concerning urinary continence, we also found a wide range of outcomes. While the used definition was identical in eight studies (0–1 pad/day), we found urinary continence rates ranging from 21.9% to 90% among the 13 included studies. Here, the caveats of heterogeneous outcomes definition are mentionable, as e.g., the study with the highest urinary continence rate [34] did not report on their used definition. Furthermore, functional outcomes are also dependent on the surgeon′s experience and expertise.

As shown above, several clinical and pathologic risk factors are associated with oncologic outcomes of SRP patients. Since careful patient selection for SRP is crucial, several studies addressed this question in detail. According to the findings of our review, the most important pre-operative prognostic factors seem to be the PSA-level and the biopsy GS before SRP, while after SRP, the pathological stage and pathologic positive LN were also of significance for oncologic outcomes. All four, i.e., a high pre-surgical PSA, the biopsy GS, the pathological stage, and LN involvement after SRP, correlated with PSM-rate, BCR-rates, clinical progression, CSS, and OS. Although the conclusions of those findings resulted from a few retrospective studies with sometimes small patient cohorts, these factors seem to be important for ideal patient selection in case of attempted local surgery with curative intent. This may also imply that performing an extended LN dissection is mandatory while performing SRP. This is in accordance with the review published in 2012 by Chade et al. [11]. The PSA and pre-SRP biopsy GS were also the strongest predictors for oncologic outcomes in their assessment. However, in contrast to our work, the pathologic stage and pathologic positive LNs were not identified as such significant factors for the prognosis after SRP. This is likely due to studies in the last eight years which showed such an association specifically.

Several limitations weaken the informative value of our study. First of all, reporting, selection, and publication biases must be considered. Furthermore, between-study heterogeneity and the lack of standardized reporting of oncologic and functional outcomes have also to be mentioned. There are currently no randomized and only a few retrospective trials addressing the role and outcomes of SRP (for the matter of fact also for nonsurgical procedures) in the case of radio-recurrent PC. The median follow-up of recently published studies is still limited, making the results subject to follow-up bias. We did not have original data sets from each series available. Nevertheless, the overall findings suggest that SRP is a feasible and effective treatment option regarding oncologic and histopathological outcomes in well-selected patients with radio-recurrent PC.

## 5. Conclusions

In this systematic review, we found that SRP is an effective local treatment option for this heterogeneous patient cohort with acceptable oncologic, histopathologic, and functional outcomes. Pre-surgical PSA and the biopsy GS seem to be the strongest pre-SRP prognostic factors for ideal patient selection. Pathologic stage and LN status after SRP are also associated with oncologic prognosis. Despite efforts, there is still a need for high-quality data from prospective well-designed studies to help strengthen our understanding of the best standard management of patients with radio-recurrent PC.

## Figures and Tables

**Table 1:** Pathologic outcomes after salvage radical prostatectomy.

First Author/Yr	Nb. of Patients	RT Type, % (RT/BT/COMB or Other Focal)	RT + ADT, %	Median Follow-Up	PSM%	Lymph Node Involvement, %	>pT2 after SRP, %
Onol/2020 [12]	94	60.6/24.5/14.9	25.5	32	17	10.6	50
Vartolomei/2019 [35]	214	-	47.7	25.3	22	18.7	43
Metcalfe/2017 [17]	70	68.6/20/11.4	26	-	20	45.8	61.4
Kenney/2016 [18]	39	61.5/38.5/0	-	-	15.4	12.8	61.5
Mandel/2016 [19]	55	49.1/50.9/0	45	36	27	21.8	50
Bates/2015 [20]	53	62.2/26.4/11.2	-	36	18.9	26.4	51
Pearce/2014 [36]	408	89/11/0	-	-	33.7	6.2	49
Yuh/2014 [21]	51	47.1/43.1/9.8	19.6	36	31.4	78.4	51
Meeks/2013 [22]	206	66/29/5	-	-	14	21	57
Gorin/2011 [23]	24	54/46/0	58	-	45.8	13.3	54.2
Chade/2011 [5]	404	65/19/16	-	-	25	16	45
Eandi/2010 [24]	18	-	2.2	18	28	20	50
Pisters/2009 [25]	42	92.9/7.1/0	0	96	-	-	-
Leonardo/2009 [26]	32	100/0/0	0	35	34.4	0	46.9
Paparel/2008 [27]	146	-	-	45	16	13	63
Sanderson/2006 [28]	51	59/23/18	18	84	35.5	15.7	44
Bianco/2005 [37]	100	29/42/29	16	60	21	9	65
Amling/1999 [29]	108	98/2/0	0	-	36	18	61
Tefilli/1998 [30]	27	-	-	34	18.5	-	33
Lerner/1995 [31]	79	90/10/0	-	50	40	8	61
Rogers/1995 [32]	40	35/65/0	2.5	39	37	5	80
Zincke/1992 [33]	32	100/0/0	0	48	-	-	-
Rainwater/1988 [34]	30	-	-	80	-	-	-

Yr = year, Nb = number, RT = radiotherapy, BT = brachytherapy, Comb = Combination therapies or other focal therapies, ADT = androgen deprivation therapy, PSM = positive surgical margins, SRP = salvage radical prostatectomy.

## Data Availability

Not applicable.

## References

[1] Cornford P., Bellmunt J., Bolla M., Briers E., Santis M.D., Gross T., Henry A.M., Joniau S., Lam T.B., Mason M.D. (2017). EAU-ESTRO-SIOG Guidelines on Prostate Cancer. Part II: Treatment of Relapsing, Metastatic, and Castration-Resistant Prostate Cancer. Eur. Urol..

[2] Agarwal P.K., Sadetsky N., Konety B.R., Resnick M.I., Carroll P.R. (2008). (CaPSURE) C of the PSURE. Treatment failure after primary and salvage therapy for prostate cancer: Likelihood, patterns of care, and outcomes. Cancer.

[3] Shariat S.F., Kattan M.W., Vickers A.J., Karakiewicz P.I., Scardino P.T. (2009). Critical review of prostate cancer predictive tools. Future Oncol..

[4] Walz J., Gallina A., Perrotte P., Jeldres C., Trinh Q.-D., Hutterer G.C., Traumann M., Ramírez A., Shariat S.F., McCormack M. (2007). Clinicians are poor raters of life-expectancy before radical prostatectomy or definitive radiotherapy for localized prostate cancer. BJU Int..

[5] Chade D.C., Shariat S.F., Cronin A.M., Savage C.J., Karnes R.J., Blute M.L., Briganti A., Montorsi F., van der Poel H.G., Van Poppel H. (2011). Salvage Radical Prostatectomy for Radiation-recurrent Prostate Cancer: A Multi-institutional Collaboration. Eur. Urol..

[6] Heidenreich A., Richter S., Thüer D., Pfister D. (2010). Prognostic Parameters, Complications, and Oncologic and Functional Outcome of Salvage Radical Prostatectomy for Locally Recurrent Prostate Cancer after 21st-Century Radiotherapy. Eur. Urol..

[7] Gotto G.T., Yunis L.H., Vora K., Eastham J.A., Scardino P.T., Rabbani F. (2010). Impact of Prior Prostate Radiation on Complications After Radical Prostatectomy. J. Urol..

[8] Moschini M., Zaffuto E., Karakiewicz P., Mattei A., Gandaglia G., Fossati N., Montorsi F., Briganti A., Shariat S.F. (2018). The effect of androgen deprivation treatment on subsequent risk of bladder cancer diagnosis in male patients treated for prostate cancer. World J. Urol..

[9] Gandaglia G., Sun M., Popa I., Schiffmann J., Trudeau V., Shariat S.F., Trinh Q.-D., Graefen M., Widmer H., Saad F. (2015). Cardiovascular Mortality in Patients With Metastatic Prostate Cancer Exposed to Androgen Deprivation Therapy: A Population-Based Study. Clin. Genitourin. Cancer.

[10] Kluth L.A., Shariat S.F., Kratzik C., Tagawa S., Sonpavde G., Rieken M., Scherr D., Pummer K. (2013). The hypothalamic–pituitary–gonadal axis and prostate cancer: Implications for androgen deprivation therapy. World J. Urol..

[11] Chade D.C., Eastham J., Graefen M., Hu J.C., Karnes R.J., Klotz L., Montorsi F., van Poppel H., Scardino P.T., Shariat S.F. (2012). Cancer Control and Functional Outcomes of Salvage Radical Prostatectomy for Radiation-recurrent Prostate Cancer: A Systematic Review of the Literature. Eur. Urol..

[12] Onol F.F., Bhat S., Moschovas M., Rogers T., Ganapathi H., Roof S., Rocco B., Patel V. (2020). Comparison of outcomes of salvage robot-assisted laparoscopic prostatectomy for post-primary radiation vs focal therapy. BJU Int..

[13] Jansen B.H., van Leeuwen P.J., Wondergem M., van der Sluis T.M., Nieuwenhuijzen J.A., Knol R.J., van Moorselaar R.J., van der Poel H.G., Oprea-Lager D.-E., Vis A.N. (2020). Detection of Recurrent Prostate Cancer Using Prostate-specific Membrane Antigen Positron Emission Tomography in Patients not Meeting the Phoenix Criteria for Biochemical Recurrence After Curative Radiotherapy. Eur. Urol. Oncol..

[14] Grubmüller B., Baltzer P., D’Andrea D., Korn S., Haug A., Hacker M., Grubmüller K.H., Goldner G.M., Wadsak W., Pfaff S. (2017). 68Ga-PSMA 11 ligand PET imaging in patients with biochemical recurrence after radical prostatectomy–diagnostic performance and impact on therapeutic decision-making. Eur. J. Nucl. Med. Mol. Imaging.

[15] Mohler J.L., Antonarakis E.S., Armstrong A.J., D’Amico A.V., Davis B.J., Dorff T., Eastham J.A., Enke C.A., Farrington T.A., Higano C.S. (2019). Prostate Cancer, Version 2.2019, NCCN Clinical Practice Guidelines in Oncology. JNCCN.

[16] Liberati A., Altman D.G., Tetzlaff J., Mulrow C., Gøtzsche P.C., Ioannidis J.P.A., Clarke M., Devereaux P.J., Kleijnen J., Moher D. (2009). The PRISMA statement for reporting systematic reviews and meta-analyses of studies that evaluate health care interventions: Explanation and elaboration. J. Clin. Epidemiol..

[17] Metcalfe M.J., Troncoso P., Guo C.C., Chen H.-C., Bozkurt Y., Ward J.F., Pisters L.L. (2017). Salvage prostatectomy for post-radiation adenocarcinoma with treatment effect: Pathological and oncological outcomes. Can. Urol. Assoc. J..

[18] Kenney P.A., Nawaf C.B., Mustafa M., Wen S., Wszolek M.F., Pettaway C.A., Ward J., Davis J.W., Pisters L.L. (2016). Robotic-assisted laparoscopic versus open salvage radical prostatectomy following radiotherapy. Can. J. Urol..

[19] Mandel P., Steuber T., Ahyai S., Kriegmair M., Schiffmann J., Boehm K., Heinzer H., Michl U., Schlomm T., Haese A. (2015). Salvage radical prostatectomy for recurrent prostate cancer: Verification of European Association of Urology guideline criteria. BJU Int..

[20] Bates A.S., Samavedi S., Kumar A., Mouraviev V., Rocco B., Coelho R., Palmer K., Patel V.R. (2015). Salvage robot assisted radical prostatectomy: A propensity matched study of perioperative, oncological and functional outcomes. European journal of surgical oncology. EJSO.

[21] Yuh B., Ruel N., Muldrew S., Mejia R., Novara G., Kawachi M., Wilson T. (2014). Complications and outcomes of salvage robot-assisted radical prostatectomy: A single-institution experience. BJU Int..

[22] Meeks J.J., Walker M., Bernstein M., Eastham J.A. (2013). Seminal vesicle involvement at salvage radical prostatectomy. BJU Int..

[23] Gorin M.A., Manoharan M., Shah G., Eldefrawy A., Soloway M.S. (2011). Urological Oncology Salvage open radical prostatectomy after failed radiation therapy: A single center experience. Cent. Eur. J. Urol..

[24] Eandi J.A., Link B.A., Nelson R.A., Josephson D.Y., Lau C., Kawachi M.H., Wilson T.G. (2010). Robotic Assisted Laparoscopic Salvage Prostatectomy for Radiation Resistant Prostate Cancer. J. Urol..

[25] Pisters L.L., Leibovici D., Blute M., Zincke H., Sebo T.J., Slezak J.M., Izawa J., Ward J., Scott S.M., Madsen L. (2009). Locally Recurrent Prostate Cancer After Initial Radiation Therapy: A Comparison of Salvage Radical Prostatectomy Versus Cryotherapy. J. Urol..

[26] Leonardo C., Simone G., Papalia R., Franco G., Guaglianone S., Gallucci M. (2009). Salvage radical prostatectomy for recurrent prostate cancer after radiation therapy. Int. J. Urol. Off. J. Jpn. Urol. Assoc..

[27] Paparel P., Cronin A.M., Savage C., Scardino P.T., Eastham J.A. (2009). Oncologic Outcome and Patterns of Recurrence after Salvage Radical Prostatectomy. Eur. Urol..

[28] Sanderson K.M., Penson D., Cai J., Groshen S., Stein J.P., Lieskovsky G., Skinner D.G. (2006). Salvage Radical Prostatectomy: Quality of Life Outcomes and Long-Term Oncological Control of Radiorecurrent Prostate Cancer. J. Urol..

[29] Amling C.L., E Lerner S., Martin S.K., Slezak J.M., Blute M.L., Zincke H. (1999). Deoxyribonucleic acid ploidy and serum prostate specific antigen predict outcome following salvage prostatectomy for radiation refractory prostate cancer. J. Urol..

[30] Tefilli M.V., Gheiler E.L., Tiguert R., Banerjee M., Forman J., Pontes J., Wood D.P. (1998). Salvage surgery or salvage radiotherapy for locally recurrent prostate cancer. Urology.

[31] E Lerner S., Blute M.L., Zincke H. (1995). Critical evaluation of salvage surgery for radio-recurrent/resistant prostate cancer. J. Urol..

[32] Rogers E., Ohori M., Kassabian V.S., Wheeler T.M., Scardino P.T. (1995). Salvage Radical Prostatectomy. J. Urol..

[33] Zincke H. (1992). Radical Prostatectomy and Exenterative Procedures for Local Failure after Radiotherapy with Curative Intent: Comparison of Outcomes. J. Urol..

[34] Rainwater L.M., Zincke H. (1988). Radical Prostatectomy After Radiation Therapy for Cancer of the Prostate: Feasibility and Prognosis. J. Urol..

[35] Vartolomei M.D., D’Andrea D., Chade D.C., Soria F., Kimura S., Foerster B., Abufaraj M., Mathieu R., Moschini M., Rouprêt M. (2019). Role of serum cholinesterase in patients treated with salvage radical prostatectomy. Urol. Oncol. Semin. Orig. Invest..

[36] Pearce S.M., Richards K., Patel S.G., Pariser J., Eggener S.E. (2015). Population-based analysis of salvage radical prostatectomy with examination of factors associated with adverse perioperative outcomes. Urol. Oncol. Semin. Orig. Invest..

[37] Bianco F.J., Scardino P.T., Stephenson A.J., Diblasio C.J., Fearn P.A., Eastham J.A. (2005). Long-term oncologic results of salvage radical prostatectomy for locally recurrent prostate cancer after radiotherapy. Int. J. Radiat. Oncol..

[38] Rouvière O., Vitry T., Lyonnet D. (2010). Imaging of prostate cancer local recurrences: Why and how?. Eur. Radiol..

[39] Luiting H.B., Van Leeuwen P.J., Busstra M.B., Brabander T., Van Der Poel H.G., Donswijk M., Vis A.N., Emmett L., Stricker P.D., Roobol M.J. (2019). Use of gallium-68 prostate-specific membrane antigen positron-emission tomography for detecting lymph node metastases in primary and recurrent prostate cancer and location of recurrence after radical prostatectomy: An overview of the current literature. BJU Int..

[40] Oehus A.-K., Kroeze S.G.C., Schmidt-Hegemann N.-S., Vogel M.M.E., Kirste S., Becker J., Burger I.A., Derlin T., Bartenstein P., Eiber M. (2020). Efficacy of PSMA ligand PET-based radiotherapy for recurrent prostate cancer after radical prostatectomy and salvage radiotherapy. BMC Cancer.

[41] Ingrosso G., Becherini C., Lancia A., Caini S., Ost P., Francolini G., Høyer M., Bottero M., Bossi A., Zilli T. (2020). Nonsurgical Salvage Local Therapies for Radiorecurrent Prostate Cancer: A Systematic Review and Meta-analysis. Eur. Urol. Oncol..

[42] Marra G., Karnes R.J., Calleris G., Oderda M., Alessio P., Palazzetti A., Battaglia A., Pisano F., Munegato S., Munoz F. (2021). Oncological outcomes of salvage radical prostatectomy for recurrent prostate cancer in the contemporary era: A multicenter retrospective study. Urol. Oncol. Semin. Orig. Invest..

[43] Alibhai S.M., Gogov S., Allibhai Z. (2006). Long-term side effects of androgen deprivation therapy in men with non-metastatic prostate cancer: A systematic literature review. Crit. Rev. Oncol..

[44] Philippou Y., Parker R.A., Volanis D., Gnanapragasam V.J. (2016). Comparative Oncologic and Toxicity Outcomes of Salvage Radical Prostatectomy Versus Nonsurgical Therapies for Radiorecurrent Prostate Cancer: A Meta–Regression Analysis. Eur. Urol. Focus.

[45] Valle L.F., Lehrer E.J., Markovic D., Elashoff D., Levin-Epstein R., Karnes R.J., Reiter R.E., Rettig M., Calais J., Nickols N.G. (2020). A Systematic Review and Meta-analysis of Local Salvage Therapies After Radiotherapy for Prostate Cancer (MASTER). Eur. Urol..

[46] Duchesne G.M., Woo H.H., Bassett J.K., Bowe S., D’Este C., Frydenberg M., King M., Ledwich L., Loblaw A., Malone S. (2016). Timing of androgen-deprivation therapy in patients with prostate cancer with a rising PSA (TROG 03.06 and VCOG PR 01-03 [TOAD]): A randomised, multicentre, non-blinded, phase 3 trial. Lancet Oncol..

[47] Payne H., Khan A., Chowdhury S., Davda R. (2012). Hormone therapy for radiorecurrent prostate cancer. World J. Urol..

[48] Siddiqui S.A., Boorjian S.A., Inman B., Bagniewski S., Bergstralh E.J., Blute M.L. (2008). Timing of Androgen Deprivation Therapy and its Impact on Survival After Radical Prostatectomy: A Matched Cohort Study. J. Urol..

[49] Boorjian S.A., Thompson R.H., Tollefson M.K., Rangel L.J., Bergstralh E.J., Blute M.L., Karnes R.J. (2011). Long-Term Risk of Clinical Progression After Biochemical Recurrence Following Radical Prostatectomy: The Impact of Time from Surgery to Recurrence. Eur. Urol..

[50] Brassetti A., Bollens R. (2019). Laparoscopic radical prostatectomy in 2018: 20 years of worldwide experiences, experimentations, researches and refinements. Minerva Chir..

[51] Klotz L. (2005). Active surveillance for prostate cancer: For whom?. J. Clin. Oncol..

[52] Fossati N., Willemse P.-P.M., den Broeck T.V., van den Bergh R.C.N., Yuan C.Y., Briers E., Bellmunt J., Bolla M., Cornford P., Santis M.D. (2017). The Benefits and Harms of Different Extents of Lymph Node Dissection During Radical Prostatectomy for Prostate Cancer: A Systematic Review. Eur. Urol..

